# A bibliometric analysis of alpha-synuclein in Parkinson’s disease from 2015 to 2024

**DOI:** 10.3389/fneur.2026.1712325

**Published:** 2026-03-04

**Authors:** Liangman Xiao, Shumin Lin, Yixuan Wu, Xin Liu, Danxia Gu, Jingqi Fan, Lixing Zhuang

**Affiliations:** 1 First Clinical Medical College, Guangzhou University of CM, Guangzhou, Guangdong Province, China; 2 First Affiliated Hospital of Guangzhou University of CM, Guangzhou, Guangdong Province, China; 3 Guangdong Clinical Research Academy of CM, Guangzhou, Guangdong Province, China

**Keywords:** alpha-synuclein, bibliometrics, Parkinson’s disease, PubMed, web of science core collection

## Abstract

**Objective:**

This study employs bibliometric analysis to systematically examine global research trends and map the intellectual landscape of α-synuclein (α-syn) in Parkinson’s disease (PD) from 2015 to 2024.

**Methods:**

On January 14, 2025, bibliographic data were extracted from the web of science core collection (WOSCC) and PubMed. Using CiteSpace (version 6.2.R4), VOSviewer (version 1.6.20), the Bibliometrix R package (4.4.2), and Microsoft Excel 2024, we analyzed publication trends, geographic and institutional contributions, journal distributions, author distributions, co-cited references, and keyword co-occurrences.

**Results:**

A total of 10,390 publications were included, with a steady annual growth (R^2^ = 0.8741). The United States, China, and Germany were the top contributing countries. Leading institutions included the University of Cambridge and the University of Pennsylvania. Core journals such as *Movement Disorders* and *International Journal of Molecular Sciences* exhibited significant influence. Keyword clustering highlighted research hotspots, including neurodegeneration, aggregation, oxidative stress, Lewy bodies, and emerging diagnostic technologies like RT-QuIC. Important trends were identified in immune mechanisms, exosome-mediated propagation, gut-brain axis involvement, and cross-disease mechanisms.

**Conclusion:**

The significance of α-syn in Parkinson’s disease research is growing. Future efforts should emphasize mechanistic studies, biomarker validation, and targeted therapies to advance personalized medicine in PD.

## 1 Introduction

Parkinson’s disease (PD) is a complicated neurodegenerative disease that is widely present in the human body and mostly affects elderly people. This disorder pathologically features dopaminergic neuron degeneration within the substantia nigra pars compacta, accompanied by aberrant α-synuclein (α-syn) protein deposition (1). The cardinal motor manifestations form a diagnostic triad comprising bradykinesia, muscular rigidity, and resting tremor, frequently coexisting with gait dysregulation and postural control deficits (2). Non-motor symptomatology encompasses cognitive decline, REM sleep behavior disorder (RBD), autonomic irregularities exemplified by constipation, sleep architecture disturbances, somatosensory abnormalities, and affective disorders, including depression (3). Epidemiological analyses establish aging as the predominant risk determinant, with male subjects exhibiting 40% greater disease burden across incidence, prevalence, and mortality metrics compared to females (4).

Building on these pathophysiological mechanisms, the prion-like propagation of misfolded α-syn along the gut-brain axis is increasingly recognized as a key pathogenic mechanism. Specifically, pathological α-syn species exhibit prion-like seeding behavior, initiating conformational conversion of native α-syn through templated aggregation (5). This self-propagating process facilitates retrograde transmission from the enteric nervous system through the vagus nerve to the brainstem, eventually reaching cortical regions (6). Notably, α-syn-mediated synaptic dysfunction and neural network disintegration emerge during prodromal stages, providing a neurobiological substrate for the clinical heterogeneity of PD manifestations (7).

As a quantitative methodology for evaluating scholarly impact, bibliometrics employs statistical analysis of publication patterns and knowledge dissemination dynamics (8). Through systematic assessment of publication/citation metrics, including publication volume, citation counts, citation-per-article ratio, and cited article percentage, this quantitative approach allows for metric-driven quantification of research influence across scientific domains (9). The past decade has witnessed exponential growth in α-syn-PD pathogenesis research, paralleled by expanding application of bibliometric analytics in Parkinson’s disease investigations. Paradoxically, systematic mapping of knowledge domains specifically linking α-syn pathobiology to PD progression remains conspicuously absent. To bridge this critical gap, our study implements multidimensional bibliometric profiling to delineate the evolving research topology, identify emergent scholarly foci, and forecast developmental vectors in this pivotal field.

## 2 Materials and methods

### 2.1 Data source and search strategy

**Figure 1:**
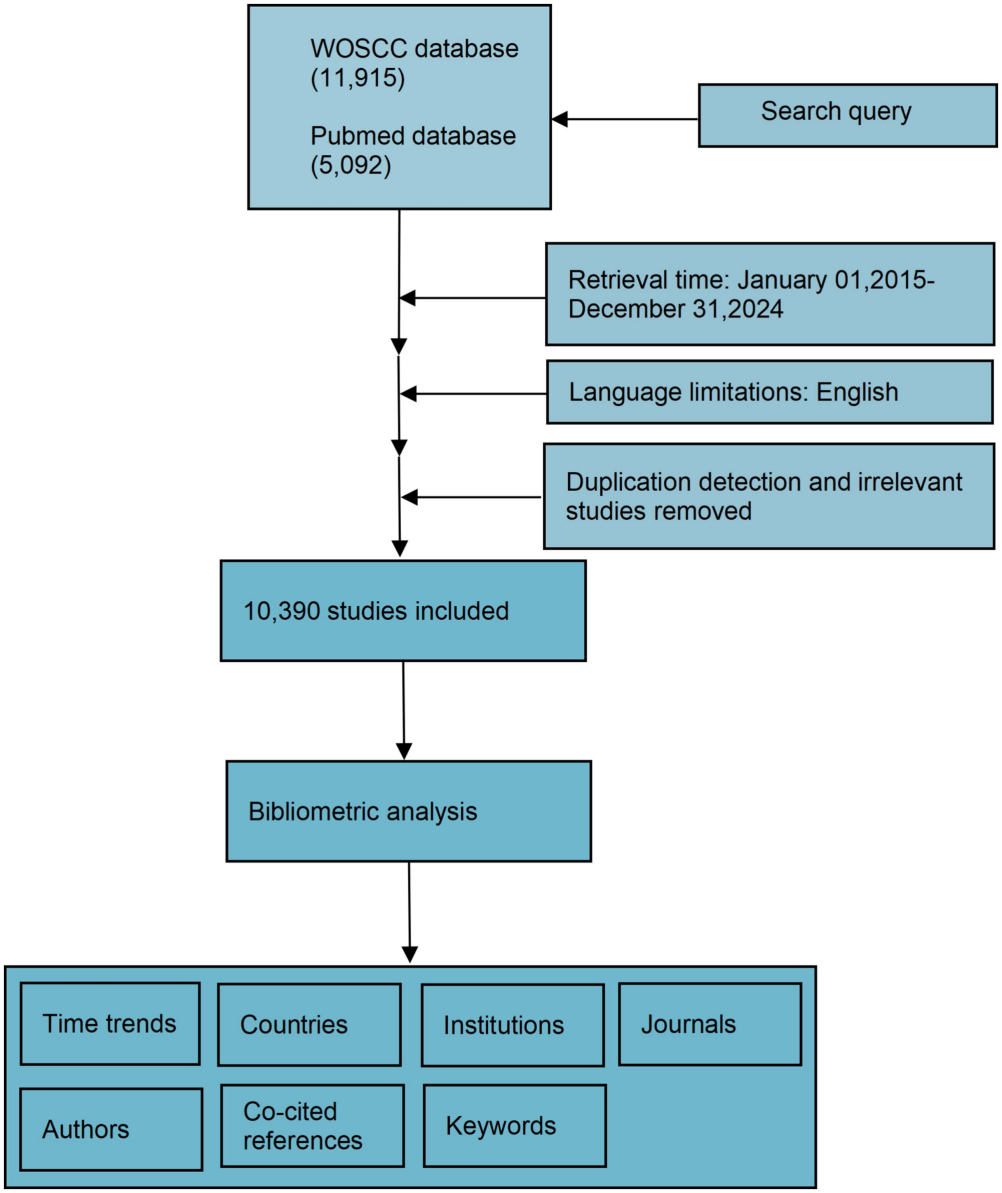
Flowchart of the selection process for the eligible literature.

We collected data from two widely used bibliometric databases, web of science core collection (WOSCC) and PubMed. To streamline data processing and minimize associated costs, a targeted search strategy was implemented using specific MeSH terms: “Parkinson’s disease” and “Alpha-synuclein.” The detailed search strategy is provided in the Supplementary Documents. Given the extensive volume of literature, manual screening was not feasible. The search strategy was carefully optimized to maximize precision. For example, in WOSCC, searches were performed separately using the title (TI), abstract (AB), and author keywords (AK) fields (10). The search was further refined to include only English-language articles and reviews (11). Through this systematic approach, 10,390 relevant references were identified following a thorough evaluation (Figure 1).

### 2.2 Data analysis

For the bibliometric analysis, CiteSpace (version 6.4.R1), VOSviewer (version 1.6.20), and the Bibliometrix R package (4.4.2) were primarily employed. PubMed records were exported in .nbib format, while WOS records were saved in plain text format (.txt). Using the built-in format conversion tool in CiteSpace (version 6.4.R1), the original PubMed files were converted to the WOS plain text format. All records were then merged into a single file and imported into CiteSpace (version 6.4.R1) for duplicate removal. After deduplication, the consolidated dataset was analyzed using VOSviewer (version 1.6.20) and Bibliometrix R package (RStudio). These tools are of crucial importance in the visualization analysis of literature. They have been widely recognized and are commonly used by numerous scholars. CiteSpace serves as an advanced computational tool for bibliometric analysis, offering powerful visualization capabilities to map the structural dynamics and evolutionary patterns of scientific knowledge across various research domains (12). VOSviewer, on the other hand, enables visual analysis of the knowledge within the research domain. It offers multiple types of view interpretations to help researchers better understand the data (13). As for Bibliometrix, it is an R package designed to gather relevant literature in the field. After that, it presents the results in a visualized form, facilitating easier comprehension and analysis (14).

## 3 Results

### 3.1 The publication trends

**Figure 2:**
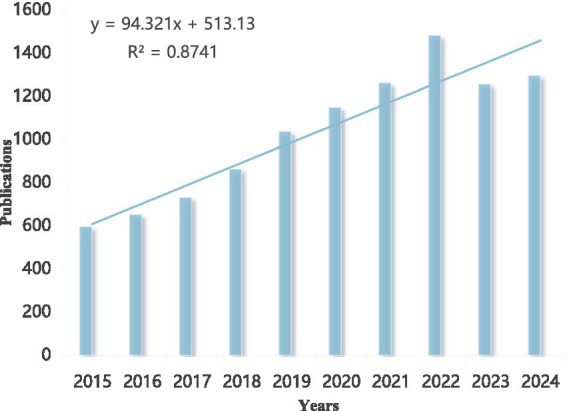
Annual publications over time in α-syn research in PD.

Over the past 10 years (2015–2024), our search identified 10,390 articles on α-syn research associated with in PD. This collection comprises 7,766 research articles and 2,624 review articles. As illustrated in Figure 2, the number of publications shows a significant and steady increasing trend over time. According to the fitted linear equation (y = 94.321x + 513.13), the annual growth is approximately 94 publications, indicating a substantial expansion in research output. The high goodness-of-fit (*R*^2^ = 0.8741) suggests a strong positive correlation between publication volume and year, reflecting a continuous rise in research activity in this field in recent years.

### 3.2 Countries and regions analysis

**Figure 3:**
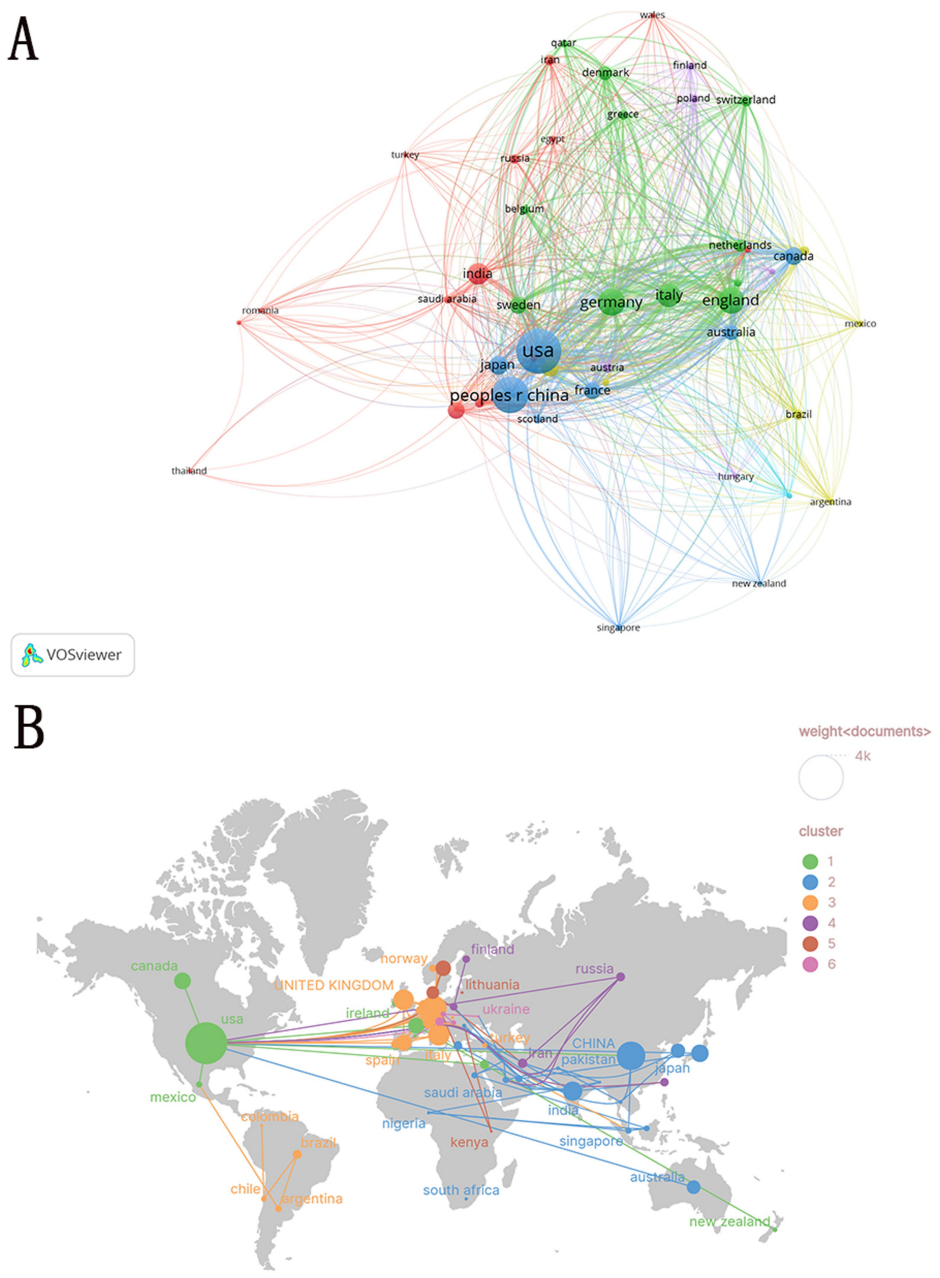
Geospatial distribution and collaborative networks of α-syn research in PD. **(A)** Worldwide research output distribution. **(B)** International collaboration patterns. (**A**: different nodes represent different countries, and the size of the nodes represents the number of publications; **B**: Node size corresponds to publication volume, The number of links indicates collaboration strength).

To delineate the global distribution of these studies, the leading contributing countries were identified based on publication output. Total Link Strength of a node is the sum of its co-occurrence strengths with all other nodes in the network, measuring both the number and intensity of its connections (15). Citations refer to the total number of times that a country’s publications on α-syn in PD research have been cited by other scholarly works. As shown in Table 1, the United States dominates the field with 3,261 publications (31.39%), followed by China (1,518, 14.61%) and Germany (1,191, 11.46%) as the most productive nations. In addition, the exceptionally high citation frequency and collaboration link strength together highlight the dual advantages of the United States in research, with outstanding research quality and a highly collaborative international cooperation model. Figure 3A, presented as a network diagram, illustrates collaboration intensity and group structures, where node size and connection density indicate each country’s significance and collaboration frequency. This visualization underscores the pivotal roles of the United States, China, and Germany, alongside collaborative clusters formed by European and Asian nations. Figure 3B depicts a geographically distributed collaboration network, emphasizing strong connections among North American, European, and Asian countries. It is noteworthy that although China ranks second globally in terms of publication output, its total link strength remains relatively low at 405, indicating a need to enhance international collaboration.

**Table 1:** Countries leading in α-syn research in PD.

Rank	Countries	Counts	Citations	Total link strength
1	USA	3,261	96,037	1,583
2	China	1,518	39,421	405
3	Germany	1,191	32,297	977
4	Italy	850	37,497	609
5	England	778	21,054	789
6	India	692	10,281	137
7	Japan	561	10,757	230
8	Canada	523	13,038	491
9	Spain	472	9,999	428
10	France	461	12,001	477

### 3.3 Affiliation analysis

**Figure 4:**
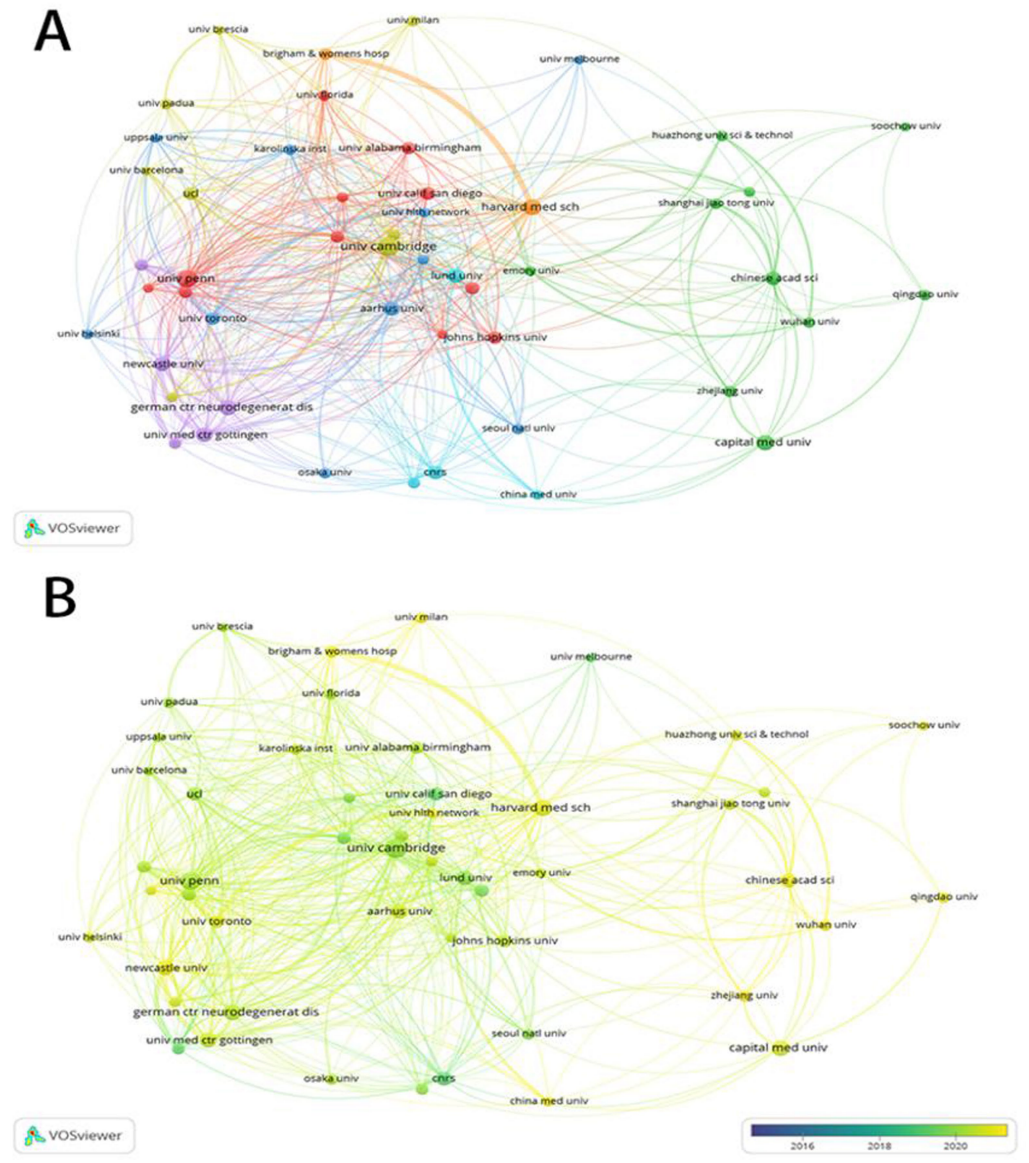
Institutional visualization analysis of α-syn research in PD. **(A)** Analysis of cooperation between institutions. **(B)** Collaborative relationships between institutions overlaid on a timeline visualization map. (**A**: Nodes represent institutions, sized by their publication count. **B**: Nodes represent institutions, sized by their publication count. Node color represents the average publication year of an institution, darker = earlier engagement, lighter/yellower = more recent engagement).

Our analysis reveals that the leading institutions by publication volume are the University of Cambridge (210), University of Pennsylvania (176), and Harvard University (151) (Table 2). The leading research institutions demonstrate a distinct geographical distribution, with two representatives each from the USA, the UK, and Germany, complemented by single contributors from China, France, Sweden, and Denmark. For further analysis, the institutions’ collaboration network based on their publication volume was screened (Figure 4A). In terms of collaborative strength, Newcastle University exhibited the most robust network, with the highest cumulative link strength, signifying the most intense collaborative interactions with other affiliations. Figure 4B presents an institutional collaboration network generated by VOSviewer, where node colors correspond to the average publication year of each institution. Dark-colored nodes, such as University of Cambridge and University Gottingen Hospital indicate earlier entry into this research field, while light-yellow nodes, such as Zhejiang University and Capital Medical University started later. A comparison of the total link strength across institutions reveals that Capital Medical University in China has a total link strength of only 7. This indicates that despite its high number of publications, the university lacks extensive collaborative relationships with other institutions.

**Table 2:** Institutions leading in α-syn research in PD.

Institutions	Country	Articles	Citations	Total link strength
University of Cambridge	UK	210	10,924	69
University of Pennsylvania	USA	176	8,667	57
Harvard University	USA	151	5,610	44
German Center For Neurodegenerative Diseases Dzne	German	134	5,405	74
Capital Medical University	China	129	2,535	7
Newcastle University	UK	126	4,658	117
Lund University	Sweden	118	5,672	41
University Gottingen Hospital	German	117	4,491	111
Cnrs	France	111	5,234	27
Aarhus University	Denmark	106	3,556	45

### 3.4 Source journal

**Figure 5:**
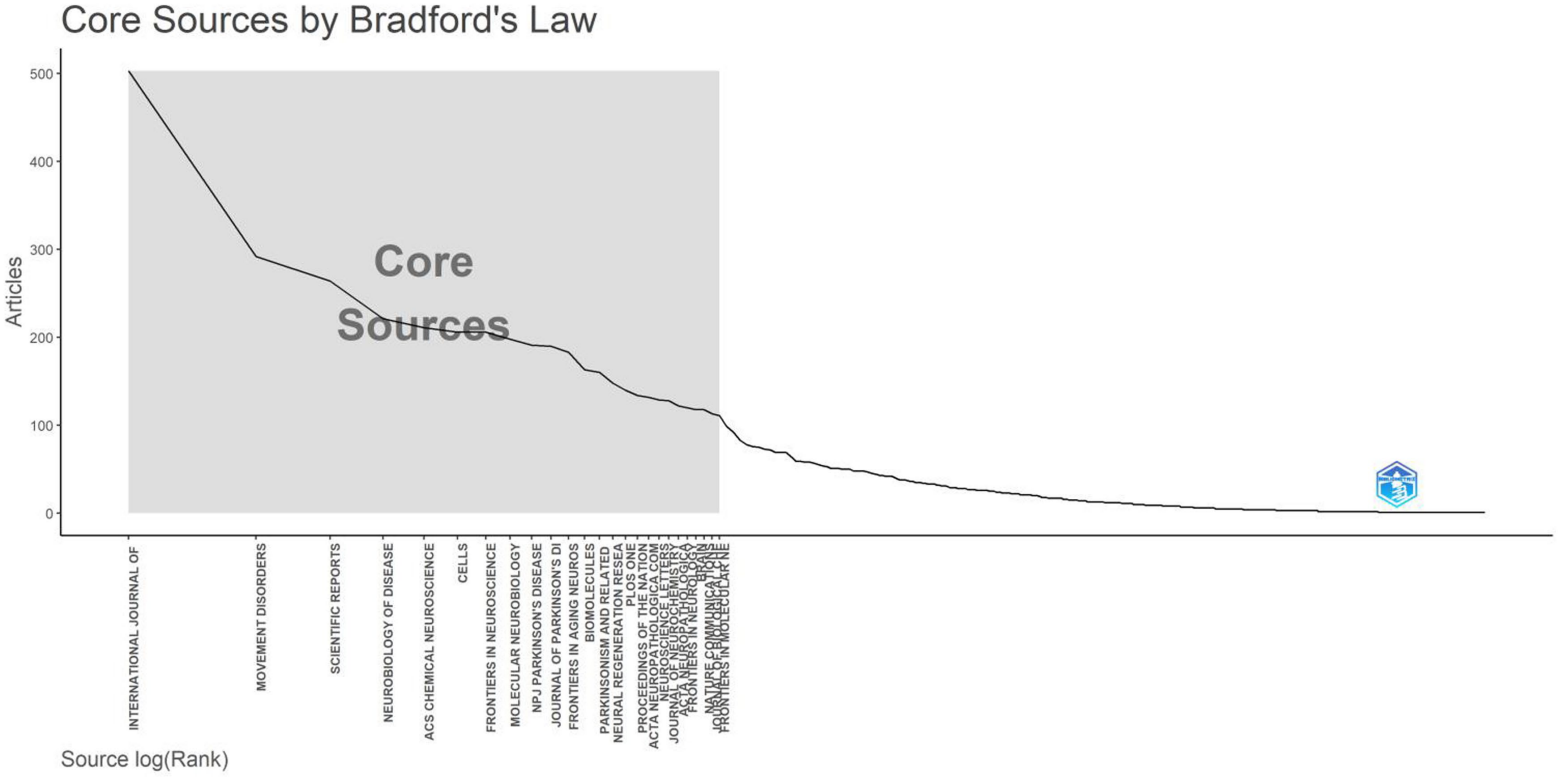
Core journal distribution patterns based on the principles of Bradford’s law.

Applying Bradford’s law analysis, a method widely recognized in the field of information science for identifying core journals within a specific domain, we identified 25 pivotal journals in the research area of α-syn and PD (Figure 5). These core journals are predominantly situated within the realms of neurology and biology, aligning with the interdisciplinary nature of PD research. Table 3 lists the leading journals in the field, presenting key metrics including aggregate publication volume, H-index values, impact factors (IF), total citations, and Journal Citation Reports data. The number of papers published in *International Journal of Molecular Sciences* (503) ranked first, far exceeding the number of second-place journals *Movement Disorders* (292). However, *Movement Disorders* has the highest citation and IF, reflecting its greater influence and the higher relevance for subsequent research.

**Table 3:** Top 10 most productive publication sources in α-syn research in PD.

Rank	SO	Counts	H-index	Total citations	IF (2024)	JCR quantile
1	International Journal of Molecular Sciences	503	36	5,435	4.9	Q1
2	Movement Disorders	292	58	10,796	7.4	Q1
3	Scientific Reports	264	47	6,251	3.8	Q1
4	Neurobiology of Disease	221	47	6,620	5.1	Q1
5	ACS Chemical Neuroscience	211	30	3,098	4.2	Q2
6	Cells	206	38	2,027	5.2	Q2
7	Frontiers In Neuroscience	206	42	5,600	3.2	Q2
8	Molecular Neurobiology	130	32	4,493	4.6	Q1
9	NPJ Parkinsons Disease	191	24	1,865	6.7	Q1
10	Journal of Parkinsons Disease	190	36	5,119	4	Q2

### 3.5 Analysis of influential authors

**Figure 6:**
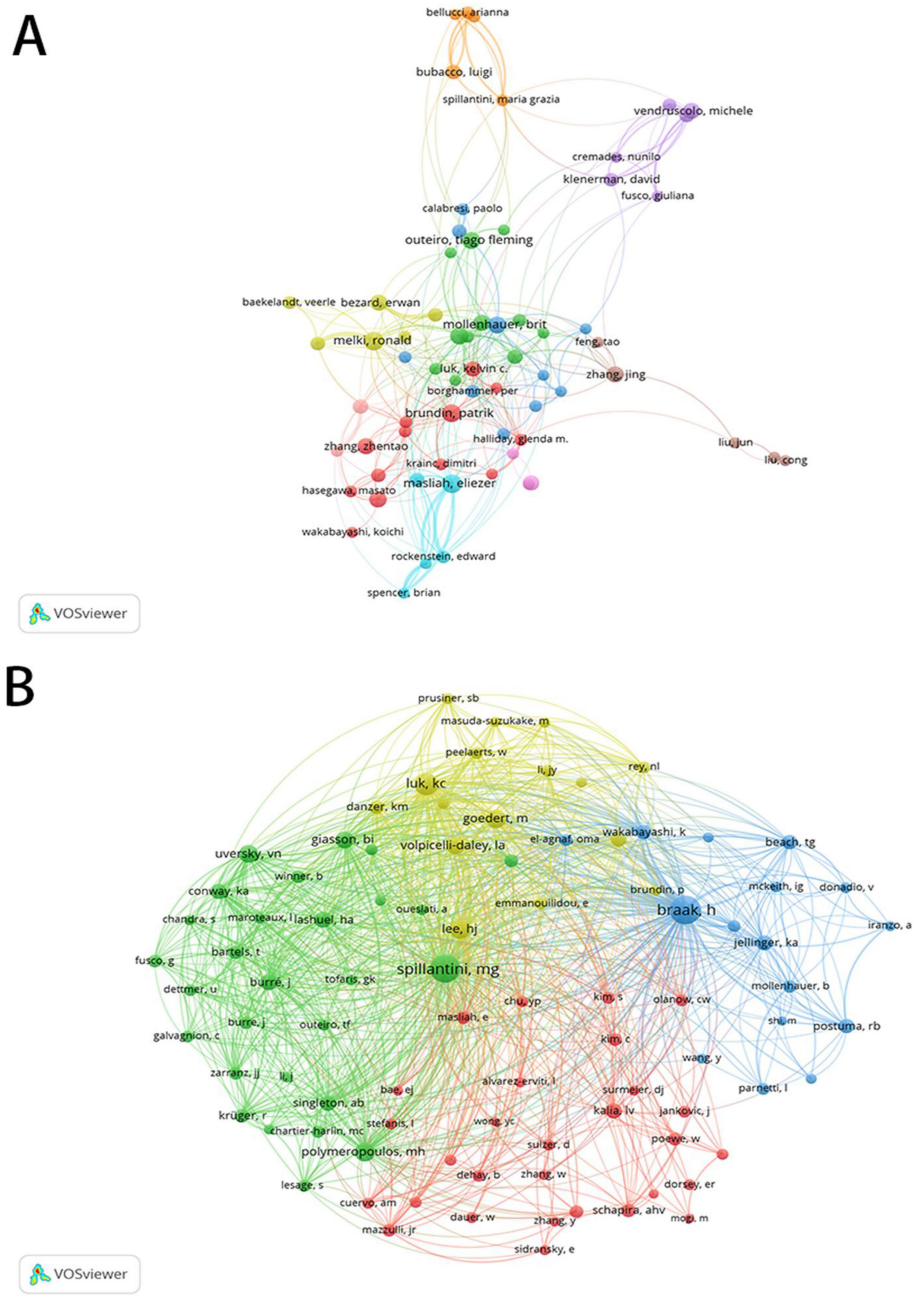
Analysis of authors in α-syn research in PD. **(A)** Visual depiction of the primary collaborative network among authors. **(B)** Visual depiction of the co-citation network among authors within the correlation framework. (**A**: Larger nodes represent more prolific authors, clusters reveal established research teams. **B**: The size of an author’s node indicates their co-citation frequency, The colors of the nodes represent different clusters of authors with similar research interests or frequent collaboration).

Figure 6 presents the author collaboration network, which offers valuable insights into the identification of potential research partners and prominent figures within the industry. As shown in Table 4 and Figure 6A, a stable core group of authors has emerged in the field of α-syn and PD research, with scholars from Europe and America leading in high-quality academic output. Among them, Masliah E stands out as the most prolific author, having published 86 articles that have garnered a total of 2,809 citations. As shown in Table 4 and Figure 6B, quantitative analysis of author co-citations indicates that more than 10 authors maintain citation records above 1,000 citations. The co-citation analysis revealed Braak H as the most frequently cited author (3,222), with Spillantini MG ranking second (3,048).

**Table 4:** The leading contributors and most frequently co-cited authors in α-syn research in PD.

Rank	Authors	Counts	Citations	Total link strength	Co-Cited authors	Citations	Total link strength
1	Masliah E	86	2,809	61	Braak H	3,222	32,614
2	Melki R	80	2,588	76	Spillantini MG	3,048	28,459
3	Brundin P	78	2,779	15	Luk KC	1,837	22,497
4	Mollenhauer B	71	2,218	26	Lee HJ	1,411	17,702
5	Outeiro TF	68	1,540	9	Polymeropoulos MH	1,385	15,020
6	Stefanis L	68	1,554	22	Goedert M	1,303	13,522
7	Hattori N	66	1,136	12	Uversky VN	1,191	12,501
8	Vendruscolo M	66	3,135	50	Volpicelli-daley LA	1,172	13,708
9	Zhang J	63	1,649	14	Giasson BI	1,102	12,745
10	Zhang Z	62	1,082	2	Burre J	1,028	12,648

### 3.6 Analysis of co-cited references and reference bursts

**Figure 7:**
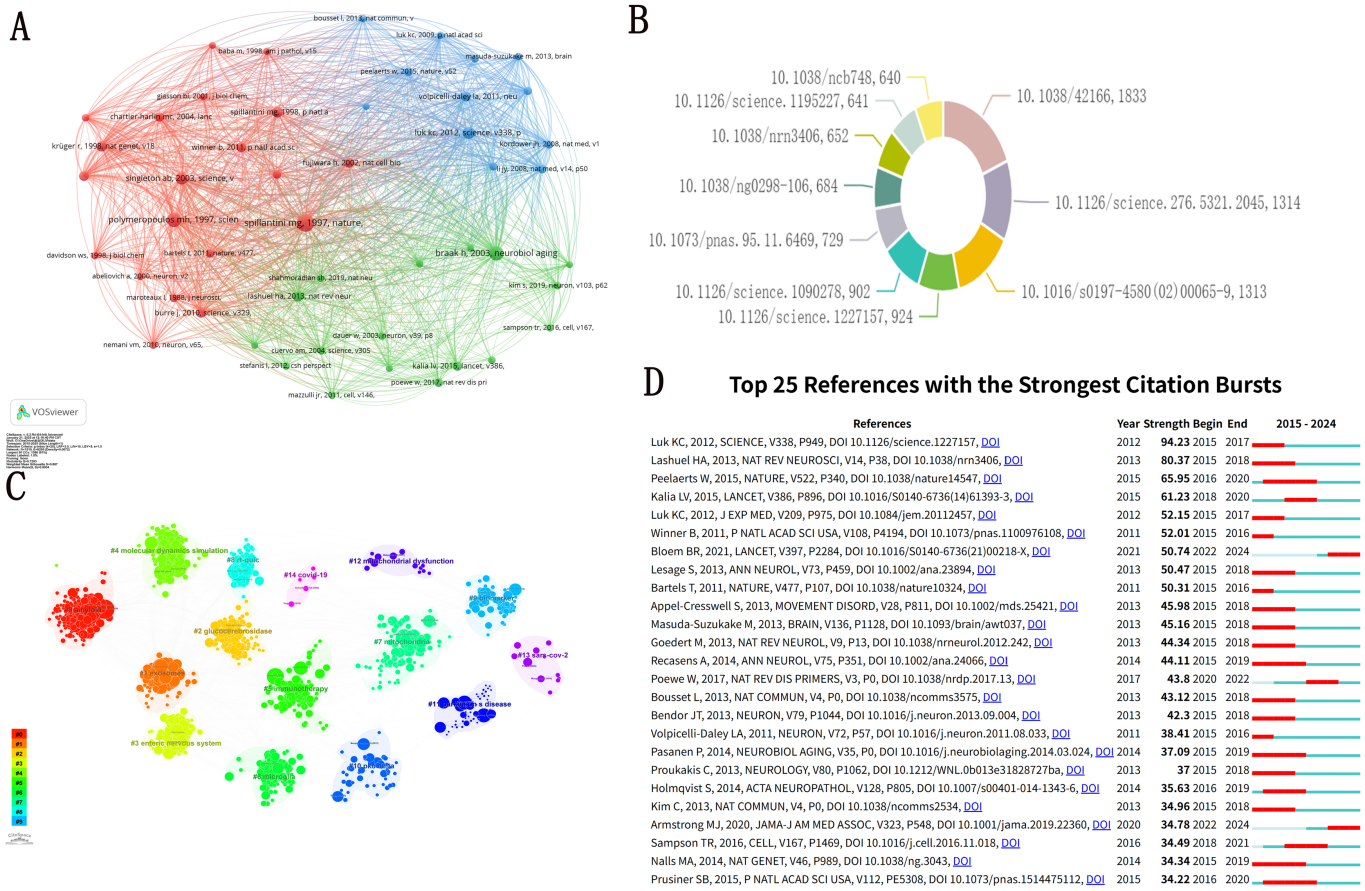
Analysis of co-cited references in α-syn research in PD. **(A)** Visualization of references co-occurrence networks. **(B)** Top 10 co-cited references. **(C)** Analysis of co-citation clusters within the literature. **(D)** The 25 most prominent references exhibiting the strongest citation bursts.

Co-cited references form the basis of a research field by identifying the core knowledge base and intellectual structure that underpin the development of scientific inquiry (16). Figure 7A presents the co-citation network of references. Figure 7B displays the top 10 co-cited references based on citation frequency. Co-citation analysis identified “Alpha-synuclein in Lewy bodies” as the most influential work (1,833). This was followed by “Mutation in the alpha-synuclein gene identified in families with Parkinson’s disease” (1,314) and “Staging of brain pathology related to sporadic Parkinson’s disease” (1,313).

The clusters depicted in various colors represent distinct research topics, with each node within a cluster indicating literature that has been co-cited. The lines connecting the nodes illustrate the co-citation relationships between the literature pieces. For instance, the red cluster labeled as #0 may pertain to research associated with “Amyloid,” while the blue cluster labeled as #1 could be linked to “Exosomes” (Figure 7C).

Reference bursts, which measure citation activity over a particular period, have become a crucial metric for understanding the dynamics of scientific research and identifying emerging trends. Figure 7D illustrates the knowledge landscape with the three most highly cited articles in recent years: the 2020 review “Diagnosis and Treatment of Parkinson Disease: A Review,” the 2017 review “Parkinson Disease” and the 2021 review of the same title, “Parkinson Disease.”

### 3.7 Keywords analysis

**Figure 8:**
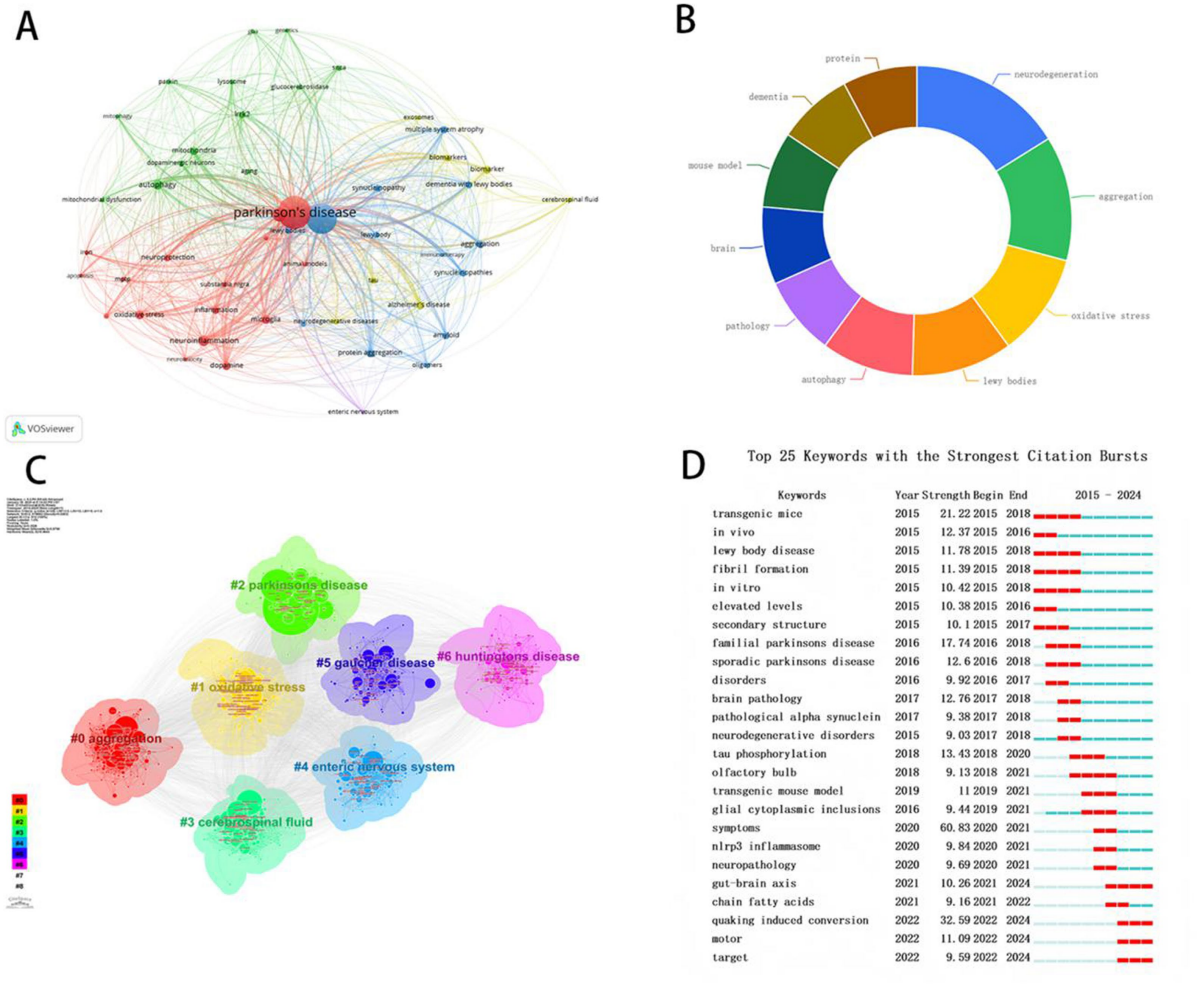
Analysis of keywords in α-syn research in PD. **(A)** Visualization of keyword co-occurrence networks. **(B)** Top 10 keywords with the largest occurrence times. **(C)** Cluster-based categorization of keywords. **(D)** Top 25 keywords with the strongest citation.

Keyword analysis serves as a valuable tool for detecting high-impact research hotspots and core themes within a research field. Figure 8A shows the keyword co-occurrence network. Figure 8B displays the top 10 most frequently occurring keywords. In the field of α-syn and PD studies prominent keywords encompass neurodegeneration (1,133 times) aggregation (1,040 times) oxidative stress (836 times) and Lewy bodies (768 times) and autophagy (705 times).

Recent keyword cluster analysis reveals that the research topics of “aggregation” and “oxidative stress” currently form prominent clusters and continue to attract substantial attention from the academic community (Figure 8C).

Keywords exhibiting the strongest bursts signify phrases that appear with high frequency within a relatively short time frame thereby reflecting temporal shifts in research focus. Burst analysis identifies 25 keywords exhibiting strong citation bursts including “transgenic mice” (strength = 21.22) “*in vivo*” (strength = 12.37) and “lewy body disease” (strength = 11.78) (Figure 8D). Additionally “quaking induced conversion” “motor” and “target” are recognized as emerging research frontiers and the current hot research topics over the past 2 years.

## 4 Discussion

### 4.1 Analysis of basic information

An analysis of publication output trends reveals a consistent growth in annual research output in the field of PD and α-syn since 2015. The fitted curve clearly demonstrates a steady upward trajectory in the number of publications over time, indicating strong research momentum and ample potential for future exploration in this area.

PD has attracted global attention as a major public health challenge, driving active international academic exchange and collaboration. Significant contributions have been made particularly by countries and institutions across Europe, North America, and East Asia. As the leading institution identified by our analysis, The University of Cambridge demonstrates the highest academic influence, excelling in publications, citations, and H-index, and maintains close collaborations with Harvard University, Imperial College London, and the University of Pennsylvania. Its research on PD encompasses basic to clinical studies, particularly focusing on α-syn aggregation mechanisms (17), immune responses (18), biomarkers (19), and gene–environment interactions (20). These investigations provide novel insights and methodologies for early diagnosis and treatment. Notably, studies on the toxicity of small α-synuclein aggregates offer critical evidence for developing future therapeutic strategies, highlighting significant clinical potential (17).

Among the various metrics for assessing journal impact, IF is a critical indicator. Studies by Zimmerman et al. (21) and Atallah et al. (22), utilizing data from the Journal Citation Reports (JCR), have demonstrated the efficacy of IF in evaluating the academic standing and dissemination influence of journals. In the field of α-syn and PD research, six out of the top 10 journals are classified in the JCR Q1 category, while the remaining four belong to Q2, collectively reflecting a high overall level of academic quality and research impact.

According to our bibliometric analysis, Masliah E is a prominent scientist in PD and α-syn research, who has made seminal contributions to the field of PD and α-syn research, focusing primarily on the development of immunotherapy strategies, elucidation of pathological mechanisms, and the establishment of animal models of disease. His team developed various immune-based interventions targeting α-syn, including AFFITOPE®-based vaccines and brain-penetrating single-chain antibodies (23, 24), which significantly reduced α-syn aggregation, attenuated neuroinflammation and neuronal loss, and improved behavioral deficits in transgenic mouse models. Furthermore, he extensively investigated the role of neuroimmune mechanisms in α-syn propagation, demonstrating that activation of Toll-like receptor 2 (TLR2) promotes cell-to-cell transmission of α-syn (25). In recent years, his work expanded into gene therapy approaches, utilizing siRNA and antisense oligonucleotides to systemically reduce α-syn expression and slow disease progression (26, 27). In model development, Masliah’s group established multiple reliable α-syn transgenic mice and primary neuronal models to simulate the pathological spread of α-syn and its associated neurodegeneration (28, 29), providing crucial platforms for understanding synucleinopathies and developing novel therapeutic strategies.

Among the top 10 co-cited authors, Braak H (3,222 co-citations) emerged as the most frequently co-cited researcher, whose seminal contribution was the 2009 proposal of the “dual-hit” hypothesis. This hypothesis suggests that pathogens can infiltrate the central nervous system through both the nasal cavity and gastrointestinal tract (30). He also established a pathological staging system for PD, which details the prion-like propagation of α-syn pathology from the medulla oblongata and olfactory bulb to the cerebral cortex (31). These groundbreaking works have provided novel perspectives on the dynamic progression of neurodegenerative diseases and established a standardized staging framework for the scientific community.

### 4.2 Research hotspots and trends

We performed an in-depth analysis of the PD and α-syn field by examining co-cited references, reference bursts, and keywords, thereby identifying and summarizing current research hotspots and emerging trends.

#### 4.2.1 Core pathogenic mechanisms

Analysis of co-cited references and reference bursts indicates that the intellectual foundation of this field is firmly rooted in the pathology of α-syn. The three most highly co-cited works, respectively, identified α-syn in Lewy bodies, linked its genetic mutations to familial PD, and proposed a staging hypothesis for neuropathological progression. Together, these foundational studies established the concept of “α-synucleinopathy” as a central pathological mechanism of PD. These seminal works have defined the dominant research paradigm over the past two decades, which focuses on the aggregation, propagation, and neurotoxicity of misfolded α-syn.

Keyword analysis revealed that in addition to PD and α-syn oxidative stress Lewy bodies and autophagy are also core terms. Together they form a PD pathological network centered on the abnormal aggregation of α-syn. Oxidative stress plays the role of both an initiator and a driver in the pathogenesis of PD. It is characterized by an imbalance between oxidants and antioxidants leading to the excessive accumulation of reactive oxygen species and reactive nitrogen species which subsequently induce widespread cellular damage (32). Oxidative stress not only directly promotes the oligomerization and fibrillation of α-syn but also activates microglia triggering the release of pro-inflammatory cytokines and thereby exacerbating neuroinflammation (33, 34). Additionally it induces DNA damage and mitochondrial dysfunction which can activate apoptotic pathways and ultimately lead to the death of dopaminergic neurons (35). Lewy bodies a key pathological hallmark of neurodegenerative diseases such as PD and dementia with Lewy bodies are primarily composed of abnormally aggregated α-syn. Studies indicate that the formation of Lewy bodies not only triggers the accumulation of toxic proteins within neurons but may also propagate neurodegenerative pathology through intercellular mechanisms (36). Autophagy a critical quality control mechanism within cells is responsible for clearing aberrant proteins and damaged organelles. In PD this pathway is frequently impaired (37). Dysfunctional autophagy leads to the accumulation of misfolded α-syn and defective mitochondria which in turn exacerbates oxidative stress and promotes the formation of Lewy bodies. Conversely oxidative stress can further disrupt autophagic activity creating a vicious cycle that collectively drives disease progression.

#### 4.2.2 Advances in diagnostic technologies and novel biomarkers

Based on the results of our reference bursts, the sharp rise in recent years in citations of the review “Diagnosis and Treatment of Parkinson Disease: A Review” underscores a growing emphasis on clinical translation and an urgent need for novel diagnostic and therapeutic strategies. In this context, α-syn has been extensively investigated as a biomarker for the diagnosis of PD. As shown in Figure 8C, the cluster term “cerebrospinal fluid” highlights the significance of cerebrospinal fluid (CSF) as a key source for α-syn extraction. Studies by scholars such as Forland et al. (38) and Kalia (39) have demonstrated that pathological aggregates of α-syn can be detected in CSF. This finding establishes CSF as a critical biological sample for investigating PD pathophysiology and developing therapeutic strategies.

Early research focused on topics such as “transgenic mice” and “*in vivo*.” In contrast, as shown in Figure 8D, around 2022, the keyword “real-time quaking-induced conversion” emerged, indicating that “real-time quaking-induced conversion” (RT-QuIC) has gained widespread attention as an emerging research direction. It also suggests that this technology is increasingly being recognized by the academic community as a key diagnostic tool for PD. RT-QuIC is an *in vitro* protein amplification assay that has significantly advanced the study of PD and related synucleinopathies. By detecting the aggregation and seeding activity of abnormal α-syn, RT-QuIC provides a powerful tool for early diagnosis, investigation of pathological mechanisms, and monitoring of disease progression. RT-QuIC has been extensively utilized in PD research to detect abnormal α-syn aggregation in biological samples. For instance, Manne et al. (40) first applied RT-QuIC to submandibular gland tissues from PD patients, demonstrating high sensitivity (100%) and specificity (94%) in detecting PD-related pathological changes, underscoring its potential for identifying prodromal PD. Christenson et al. (41) further enhanced detection sensitivity by developing nanoparticle-enhanced quaking-induced conversion (Nano-QuIC), enabling the detection of α-syn seeding activity in blood samples and paving the way for non-invasive PD diagnosis. Beyond tissue samples, RT-QuIC has been successfully applied to body fluid analysis. Luan et al. (42) demonstrated that RT-QuIC could detect α-syn seeding activity in saliva with a sensitivity of 76.0% and specificity of 94.4% in PD patients, offering a novel biomarker for clinical diagnosis. Kuzkina et al. (43) found that RT-QuIC detection in skin biopsies exhibited high sensitivity (97.4%) in patients with idiopathic rapid eye movement sleep behavior disorder (iRBD), suggesting its potential as an early marker for α-synucleinopathies. In terms of detection accuracy for biological samples, CSF is currently considered the gold standard for α-synuclein seed amplification assays (α-syn SAA) in diagnosing PD, owing to its high analytical performance. Skin biopsy emerges as a strong alternative, demonstrating sensitivity and specificity approaching that of CSF. Given the practical challenges associated with CSF collection, skin-based testing has become a more feasible and cost-effective option in clinical practice (44, 45). The application of RT-QuIC technology provides critical support for early diagnosis, disease subtyping, and the development of therapeutic targets, positioning it as a key advancing direction in PD diagnostics and treatment.

#### 4.2.3 The enteric nervous system: a gateway for pathological α-syn propagation in PD

The emergence of the “enteric nervous system (ENS)” as an independent cluster underscores the widespread attention and ongoing research into the hypothesis that α-syn pathology originates peripherally and propagates along the gut-brain axis. Multiple studies indicate that abnormal α-syn aggregation within the ENS is closely associated with glial cell activation and can occur in early disease stages, highlighting its potential as a window for early diagnosis (46, 47). Anatomically, the ENS connects to the brain via the vagus nerve. Its high density in the upper gastrointestinal tract, including the esophagus and stomach, closely aligns with the projection patterns of the vagus nerve, providing a structural basis for the prion-like retrograde transmission of α-syn from the gut to the brainstem. This concept is further supported by animal studies, in which intestinal administration of α-syn fibrils has been shown to induce brain pathology and neurodegeneration through vagal pathways (48). However, animal studies indicate that ENS pathology does not invariably lead to propagation to the central nervous system, suggesting that the underlying mechanisms remain incompletely understood. Further research is needed to elucidate the relationship between ENS and CNS pathology, which likely involves multifactorial interactions including immune regulation and impaired barrier function (49, 50).

Thus, the ENS serves not only as an early marker of PD pathology, but also as a promising target for understanding the mechanisms of cell-to-cell α-syn propagation and for developing early intervention strategies. Nevertheless, the relationship between ENS involvement and central nervous system pathology remains to be fully elucidated, highlighting a critical gap in future research.

#### 4.2.4 Cross-disease mechanisms and therapeutic targets

The emergence of “Gaucher’s disease” and “Huntington’s disease” in keyword clustering reflects mechanistic intersections between PD and other neurodegenerative disorders. Huntington’s disease, like PD, is a neurodegenerative disorder, and research in Huntington’s disease models has provided important insights into neuroinflammation, intercellular spread, and autophagy regulation, which are relevant to understanding the propagation mechanisms of α-syn in PD (51). The association between Gaucher disease and PD is primarily characterized by the abnormal accumulation of α-syn. In Gaucher disease, mutations lead to the loss of β-glucocerebrosidase (GCase) activity, causing metabolic disturbances that impair the normal degradation of α-syn, resulting in its intracellular accumulation and the formation of Lewy bodies. Since restoring GCase activity reduces α-syn accumulation, enhancing GCase activity is considered a potential therapeutic strategy for Gaucher disease-associated PD, offering new targets for PD treatment (52).

“Target” has emerged as a new research hotspot. Targeting α-syn has emerged as a highly promising therapeutic strategy in recent years. Various approaches have been proposed and validated. For example, Wrasidlo et al. (53) developed a novel compound that effectively ameliorated behavioral deficits in PD models, highlighting the potential of α-synuclein-targeted disease-modifying therapies. Additionally, Zhang et al. (54) targeted miR-let-7a to suppress α-synuclein-induced microglial inflammation, offering new insights into gene therapy for PD. These advancements underscore the growing emphasis on α-syn as a central therapeutic target in PD research. α-syn not only serves as a key biomarker for PD but also holds potential as a therapeutic target, offering novel strategic approaches to address this global challenge.

### 4.3 Limitations

Owing to the extensive body of relevant literature, the analysis is limited to the period from 2015 to 2024, which may omit earlier foundational research that has shaped the field. Additionally, the data were sourced exclusively from WOSCC and Pubmed and included only publications in English. These limitations may constrain our perspective and result in a narrowed scope of the available evidence. Nonetheless, these limitations do not diminish the study’s ability to illuminate the current state and trajectory of PD and α-syn research.

## 5 Conclusion

This bibliometric analysis offers a comprehensive overview of the research landscape on α-syn in PD between 2015 and 2024, highlighting major advancements, influential researchers, and emerging trends. The consistent growth in publication output reflects sustained scientific and clinical interest in α-syn as a central pathogenic factor in PD. The intellectual foundation of the field is built upon seminal studies that have elucidated the role of α-syn in Lewy body formation, genetic mutations, and neuropathological staging. Recent research trends are increasingly shifting toward translational applications, including the use of RT-QuIC for early diagnosis and the development of α-synuclein-targeted therapies. Growing interest in immune mechanisms, exosome-mediated propagation, and cross-disease pathways reflects a movement toward integrated, multidisciplinary strategies for understanding and treating PD.

Despite limitations in data scope and language, this study successfully maps the evolution of research themes, patterns of international collaboration, and the contributions of leading institutions and authors. It is clear that α-syn remains a cornerstone of PD, with ongoing efforts focused on leveraging its pathophysiological roles to improve diagnostic and therapeutic strategies. Future studies should continue to emphasize mechanistic investigation, biomarker validation, and the development of targeted interventions, ultimately advancing the pursuit of personalized medicine for PD.

## Data Availability

The original contributions presented in the study are included in the article/Supplementary material, further inquiries can be directed to the corresponding author.
