# Supplementary material for: A bibliometric analysis of alpha-synuclein in Parkinson’s disease from 2015 to 2024
Source: Front Neurol. 2026 Mar 4;17:1712325. doi: 10.3389/fneur.2026.1712325 (PMC12995758; doi:10.3389/fneur.2026.1712325)
Supplement: Supplementary file 1 [file Table_1.DOCX]

**Supplement Document 1: Mesh Terms and Search Strategy**

Literature data were collected from the Science Citation Index Expanded (SCIE). To ensure comprehensiveness, the search subject terms were identified using MeSH, with the final search strategy being #1 AND #2.

#1((TI=(“alpha Synuclein” OR “Non-AB Component of AD Amyloid Protein” OR “Non AB Component of AD Amyloid Protein”)) OR AB=(“alpha Synuclein” OR “Non-AB Component of AD Amyloid Protein” OR “Non AB Component of AD Amyloid Protein”)) OR AK=(“alpha Synuclein” OR “Non-AB Component of AD Amyloid Protein” OR “Non AB Component of AD Amyloid Protein”)

#2((TI=(“Parkinson's disease” OR “Idiopathic Parkinson's Disease” OR “Lewy Body Parkinson's Disease” OR “Parkinson's Disease, Idiopathic” OR “Parkinson's Disease, Lewy Body” OR “Paralysis Agitans” OR “Parkinson's Disease” OR “Idiopathic Parkinson Disease” OR “Lewy Body Parkinson Disease” OR “Primary Parkinsonism” OR “Parkinsonism, Primary” OR “Parkinson Disease, Idiopathic”)) OR AB=(“Parkinson's disease” OR “Idiopathic Parkinson's Disease” OR “Lewy Body Parkinson's Disease” OR “Parkinson's Disease, Idiopathic” OR “Parkinson's Disease, Lewy Body” OR “Paralysis Agitans” OR “Parkinson's Disease” OR “Idiopathic Parkinson Disease” OR “Lewy Body Parkinson Disease” OR “Primary Parkinsonism” OR “Parkinsonism, Primary” OR “Parkinson Disease, Idiopathic”)) OR AK=(“Parkinson's disease” OR “Idiopathic Parkinson's Disease” OR “Lewy Body Parkinson's Disease” OR “Parkinson's Disease, Idiopathic” OR “Parkinson's Disease, Lewy Body” OR “Paralysis Agitans” OR “Parkinson's Disease” OR “Idiopathic Parkinson Disease” OR “Lewy Body Parkinson Disease” OR “Primary Parkinsonism” OR “Parkinsonism, Primary” OR “Parkinson Disease, Idiopathic”)

The same comprehensive search strategy was also applied to the PubMed database. The search formula was as follows.

((“alpha Synuclein” OR “Non-AB Component of AD Amyloid Protein” OR “Non AB Component of AD Amyloid Protein”)[MeSH Terms]) AND ((“Parkinson's disease” OR “Idiopathic Parkinson's Disease” OR “Lewy Body Parkinson's Disease” OR “Parkinson's Disease, Idiopathic” OR “Parkinson's Disease, Lewy Body” OR “Paralysis Agitans” OR “Parkinson's Disease” OR “Idiopathic Parkinson Disease” OR “Lewy Body Parkinson Disease” OR “Primary Parkinsonism” OR “Parkinsonism, Primary” OR “Parkinson Disease, Idiopathic)[MeSH Terms])
